# Effect of Operating Conditions on Membrane Fouling in Pilot-Scale MBRs: Filaments Growth, Diminishing Dissolved Oxygen and Recirculation Rate of the Activated Sludge

**DOI:** 10.3390/membranes11070490

**Published:** 2021-06-29

**Authors:** Petros Gkotsis, Dimitra Banti, Anastasia Pritsa, Manassis Mitrakas, Petros Samaras, Efrosini Peleka, Anastasios Zouboulis

**Affiliations:** 1 Laboratory of Chemical and Environmental Technology, Department of Chemistry, Faculty of Sciences, Aristotle University of Thessaloniki, GR-54124 Thessaloniki, Greece; petgk@chem.auth.gr (P.G.); peleka@chem.auth.gr (E.P.); 2 Laboratory of Technologies of Environmental Protection and Utilization of Food By-Products, Department of Food Science and Technology, International Hellenic University, GR-57400 Thessaloniki, Greece; bantidim@gmail.com (D.B.); samaras@ihu.gr (P.S.); 3 Analytic Chemistry Laboratory, Department of Chemical Engineering, School of Engineering, Aristotle University of Thessaloniki, GR-54124 Thessaloniki, Greece; pritsa.anastasia@gmail.com (A.P.); manasis@eng.auth.gr (M.M.)

**Keywords:** MBR, membrane fouling, filamentous microorganisms, recirculation rate, denitrification

## Abstract

This is the first study that examines the effect of operating conditions on fouling of Membrane Bio-Reactors (MBRs), which treat municipal wastewater in field conditions, with specific regard to the controlled development of filamentous microorganisms (or filaments). The novelty of the present work is extended to minimize the dissolved oxygen (DO) in recirculated activated sludge for improving the process of denitrification. For this purpose, two pilot-scale MBRs were constructed and operated in parallel: (i) Filament-MBR, where an attempt was made to regulate the growth of filaments by adjustment of DO, the Food-to-Microorganisms (F/M) ratio and temperature, and (ii) Control-MBR, where a gentle stirring tank was employed for the purpose of zeroing the DO in the recycled sludge. Results showed that low temperature (<15 °C) slightly increased the number of filaments in the Filament-MBR which, in turn, decreased the Trans-Membrane Pressure (TMP). As the Soluble Microbial Products (SMP) and the colloids are considered to be the basic foulants of membranes in MBR systems, specific attention was directed to keep their concentration at low values in the mixed liquor. The low F/M ratio in the aeration tanks which preceded the membrane tank was achieved to keep the SMP proteins and carbohydrates at very low values in the mixed liquor, i.e., less than 6 mg/L. Moreover, as a result of the low recirculation rate (2.6∙Q_in_), good aggregation of the produced excess sludge was achieved, and low concentration of colloids with a size ≤50 nm (nearly the membranes’ pore size used for filtration/separation) was measured, accounted for maximum 15% of the total colloids. Additionally, the increase in filamentous population at the Filament-MBR contributed to the further reduction of colloids in the mixed liquor at 7.9%, contributing beneficially to the reduction of TMP and of membrane fouling. The diminishing of DO in the recirculated sludge improved denitrification, and resulted in lower concentrations of Ν-NO_3_^−^ and TN in the effluent of the Control-MBR. Furthermore, the recirculation rate of Q_r_ = 2.6∙Q_in_, in comparison with Q_r_ = 4.3∙Q_in_, resulted in improved performance regarding the removal of N-NH_4_^+^. Finally, high organics removal and ammonium nitrification was observed in the effluent of both pilots, since COD and Ν-ΝH_4_^+^ concentrations were generally in the range of 10–25 mg/L and <0.1 mg/L, respectively.

## 1. Introduction

Membrane bioreactors (MBRs) have been increasingly employed in wastewater and water treatment due to their significant advantages over the conventional activated sludge process (CASP), such as superior effluent quality and low space requirement. However, membrane fouling still remains the major drawback of MBRs; therefore, several methods have been applied in fouling control and mitigation, ranging from conventional methods, such as aeration, backwashing or relaxation, to innovative methods, such as the use of several coagulants/flocculants, adsorbing agents or bio-carriers, membrane surface modification techniques, application of electric field or ultrasound, etc. [1,2,3].

In recent years, the utilization of filamentous microorganisms/bacteria has been adopted as a novel anti-fouling biological method with promising results. The presence of filamentous bacteria (or filaments), a group of microorganisms, has been identified as the main reason for the sludge foaming and bulking during treatment with the conventional activated sludge process [4]. The presence of filamentous bacteria is generally considered to be detrimental to the overall operation of MBR systems as well, especially in terms of membrane fouling. It has been reported that excessive growth of filaments changes the shape and hydrophobicity of sludge flocs and increases the amount of Extracellular Polymeric Substances (EPS), resulting in severe membrane fouling [5,6]. However, according to the study of Li et al. [7], filaments do not affect the membrane fouling rate, although they have the ability to change the bio-floc morphology.

Other researchers support the opinion that the presence of filaments improves sludge characteristics and reduces membrane fouling. Specifically, Wang et al. [8] showed that the abundance of filamentous bacteria mitigated the fouling of submerged membranes due to improved sludge characteristics, such as larger particle size distribution and lower hydrophobic content in Soluble Microbial Products (SMP), resulting in the formation of a special fouling layer on the membrane, which prevented the EPS and SMP from absorbing onto its surface or blocking its pores. However, Banti et al. [9] and Gkotsis et al. [10] observed that moderate populations of filaments reduced the membrane fouling of submerged membranes, as indicated by the decrease in Trans-Membrane Pressure (TMP) at Filament Index (FI) values of 2–3 and 1–2, respectively, and attributed the TMP decrease to the formation of sludge with high porosity and low adhesion capability at the membrane surface. All the aforementioned studies, however, correlate the development of filaments with fouling propensity in lab-scale MBRs, which treat synthetic municipal wastewater. Although lab-scale setups have low operating costs, they cannot effectively simulate full-scale installations in field conditions, especially in terms of capacity, applied operating conditions and energy requirements. In addition, the removal of nitrogen via the biological processes of nitrification/denitrification has not been sufficiently investigated in the filament-MBR systems. Studying the influence of the COD:TN ratio on sludge properties and fouling of MBR submerged membranes, Hao et al. [11] found that small quantities of filamentous microorganisms improved membrane performance, while the removal of nitrogen by the denitrification process was not investigated. You and Sue [12] also examined the effect of filaments on fouling of a membrane bioreactor from a biological standpoint, focusing mainly on the effect of the bacterial strain on TMP, flux and foaming properties.

This is the first study which aims to examine the effect of operating conditions, such as recirculation rate of activated sludge and filaments growth, on membranes’ fouling in pilot-scale MBRs that are constructed and operated under the field conditions of a municipal wastewater treatment plant. Another element of novelty in the present work is the pre-treatment of the recycled activated sludge for diminishing the DO and its effect on the process of denitrification, which has not been examined in the relevant literature. For this reason, two pilot-MBRs were constructed and operated in parallel: (i) Filament-MBR, where an attempt was made to regulate the growth of filaments by controlling dissolved oxygen (DO), food to microorganisms (F/M) and temperature, and (ii) Control-MBR, where the recycled activated sludge stream was pre-treated by gentle stirring in a specially designed tank for diminishing the DO to enhance denitrification and, thus, nitrogen removal.

## 2. Materials and Methods

### 2.1. Configuration and Operation of Pilot-Scale MBR Treatment Systems

**Figure 1:**
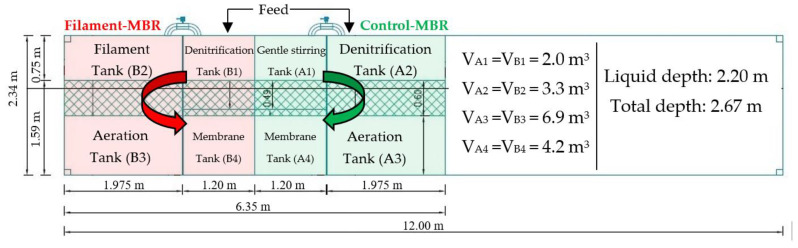
Top-view layout of the pilot-scale Membrane Bioreactor.

The membrane bioreactor system was constructed by Environmental Engineering S.A., Thessaloniki, Greece, consisting of two pilot-MBRs: (a) Control-MBR and (b) Filament-MBR (Figure 1), and is located in the region of Nea Santa, Kilkis, Greece (please see also Figure S1 in Supplementary Material). Both pilot-MBRs include a denitrification, aeration, and membrane tank. The Filament-MBR incorporates a step-aeration of two tanks (Figure 1), where the first filament tank, B2, is focusing on dissolved oxygen (DO) and on F/M ratio variation to regulate the development of filamentous microorganisms. In contrast, the Control-MBR incorporates the de-aeration tank, A1, which is placed before the denitrification tank to remove dissolved oxygen from the sludge recirculation stream to study the effect on the efficiency of the denitrification process. The construction and concurrent operation of two pilot-MBRs allowed for the better evaluation of filaments’ development under different conditions.

Both pilot-MBRs were fed with real municipal wastewater, i.e., with the effluent from the balancing tank, which was pretreated with a screen of 1 cm and rotary drum screen of 1 mm, aerated grid chamber and grease and fat removal, for a total period of approximately five months (August 2020–December 2020). The inlet wastewater was added to the denitrification tank of each pilot-MBR (Tanks A2 and B1 in Figure 1) by means of a mono-pump at the rate of Q_in_ = 700 L/h. The mixed liquor circulated in the four-tank tank sequence, following the order A1–A2–A3–A4 in the Control-MBR and the order B1–B2–B3–B4 in the Filament-MBR; a second mono-pump was used to withdraw the permeate from the upper end of each membrane at the rate of Q_out_ = 700 L/h. The sludge was recirculated from the membrane tank of each pilot-MBR (A4 and B4) to the de-aeration tank (A1) and to the denitrification tank (B1), respectively, at the rate of Q_r_ = 2.6∙Q_in_ = 1800 ± 100 L/h. However, a higher recirculation rate (Q_r_ = 4.3∙Q_in_) was also tested for a period of three weeks, in order to study the effect of the recirculation rate on the de-aeration efficiency and, thus, on the denitrification process. Level sensors were installed to control the level of the mixed liquor in the membrane tanks and avoid their draining. The permeate collection unit (installed in the engine room) was the final recipient of the produced permeate.

The air needed for the biomass, as well as for the cleaning of the applied membranes, was supplied by an air blower. The concentration of the dissolved oxygen was monitored by DO-meters in the range of 2–3 mg/L in the aeration tanks, <0.2 mg/L in the denitrification tanks and 0.5 ± 0.3 mg/L in the filament tank. In each pilot-MBR, a flat sheet of polyethersulfone ultrafiltration membrane module type NADIR^®^ UP150, Microdyn-Nadir GmbH, Wiesbaden, Germany, was used, with a pore size of 0.04 μm, a nominal membrane area of 52 m^2^ (made) and a maximum TMP filtration limit of 400 mbar. However, when the TMP reached 350 mbar, the membranes were chemically cleaned, initially with NaOCl and then with citric acid, according to the cleaning protocol provided by the membrane manufacturer. Finally, it is noteworthy that the overall operation of both pilot-MBRs (Control-MBR and Filament-MBR) was fully automated by a supervisory control and data acquisition (SCADA) system. The applied operating conditions and main membrane characteristics for both pilot-MBRs are presented in Table 1.

### 2.2. Filament Index (FI) Determination

The FI was determined according to the method proposed by Eikelboom [13] (please see also Section S1 in Supplementary Material). A Light Sheet Microscope (LSM, Observer Z1, Zeiss) was used at 50×, 100× and 200× magnification to determine the filament index (FI) of the mixed liquor samples. Analysis of the obtained images was conducted using the ZEN lite software. Following the sludge acclimatization period and the achievement of steady-state conditions in the bioreactor, more than 200 sludge images were obtained and examined for each operating phase. In this study, the most representative images were selected and presented.

### 2.3. Determination of Effluent Quality Parameters

The quality parameters of the pilot MBRs effluent, i.e., concentrations of COD, total nitrogen (TN), N-NH_4_^+^ and N-NO_3_^−^, were determined with standardized Hack–Lange LCK test kits (with part numbers 314, 238, 304, and 339, respectively), along with a DR-3900 spectrophotometer.

### 2.4. Measurement of SMP Concentration

Both the carbohydrate fraction of SMP (SMP_c_) and the protein fraction of SMP (SMP_p_) were determined. SMP_c_ were extracted by the following procedure: mixed liquor samples were obtained daily from the bioreactor and centrifuged to separate the solid biomass. Then, the phenol-sulfuric acid method [14], which is the most widely used colorimetric method for the determination of carbohydrate concentration in aqueous solutions, was applied in the supernatant for determination of the carbohydrate fraction of SMPs. The principle of this method is that carbohydrates, when dehydrated by reaction with concentrated sulfuric acid, produce furfural derivatives which react with phenol and develop a detectible colour (please see also Section S2 in Supplementary Material for more details). The concentration of proteins was measured according to the modified Lowry method in triplicates [15], while the measurement was calibrated with bovine serum albumin (BSA, Sigma Aldrich). The measurement of SMP_c_ and SMP_p_ was conducted twice a week.

### 2.5. Determination of Particle Size Distribution

Dynamic light scattering (DLS) (Brookhaven Instruments Corporation) was used to measure the particle size distribution (0–1.5 μm) for the mixed liquor samples, originating from the membrane tanks and from the effluents of two MBR lines. The respective samples were pre-filtered with syringe filters of 1.5 μm (Whatman Puradisc, Nylon) before the measurement. The results were processed by using the Brookhaven Particle Solutions Software. For the measurements of particle size distribution, two samples per week were received.

## 3. Results and Discussion

### 3.1. Fouling Examination

In this section, the fouling propensity of the employed membranes is investigated. First, the developed TMP is presented and correlated with the growth of filamentous microorganisms and the activated sludge temperature in both pilot-MBRs. Then, the evolution of Soluble Microbial Products (SMP) and colloids, which are considered to be the most significant foulants in membrane bioreactors, is presented and discussed.

#### 3.1.1. Effect of Filaments’ Development on Membrane Fouling—Correlation of TMP with FI and Temperature

In the present study, both pilot-MBRs were operated under constant filtration rate 700 L/h = 13.5 L/m^2^h (<18 L/m^2^h critical flux) and the increase of the Trans-Membrane Pressure (TMP) was employed as the main fouling index. Under constant filtration rate conditions, the evolution of TMP in a membrane bioreactor is commonly considered to consist of three subsequent stages/processes that include: (a) the initial adsorption of fouling substances (usually EPS and SMP) after the immersion of the membrane in the mixed liquor/biomass (Stage 1); (b) the long-term fouling (Stage 2), which is characterized by further adsorption and deposition of organics, colloids and bio-flocs on the membrane surface; (c) TMP jump (Stage 3), when TMP suddenly rises until it reaches the maximum limit set according to the membrane specifications.

**Figure 2:**
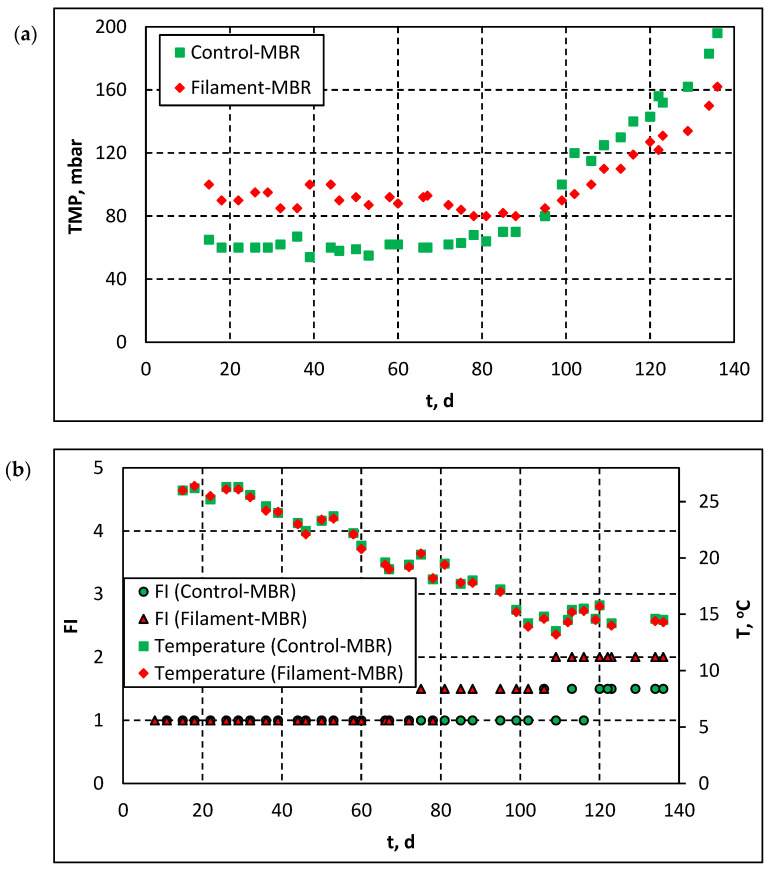

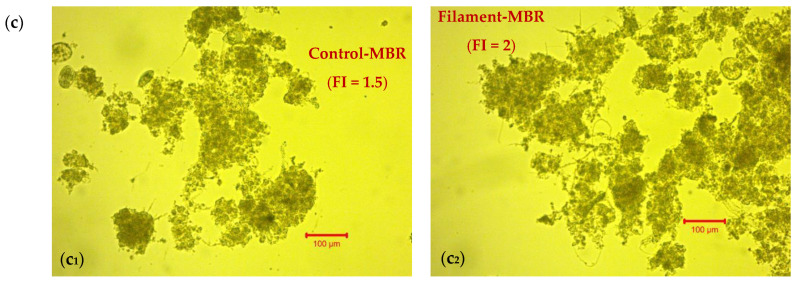
(**a**) Development of TMP during the pilot-scale MBR operation, (**b**) evolution of FI and temperature during the pilot-scale MBR operation and (**c**) indicative FI observation images with optical microscopy (134th day of operation of the pilot-scale MBR).

**Figure 3:**
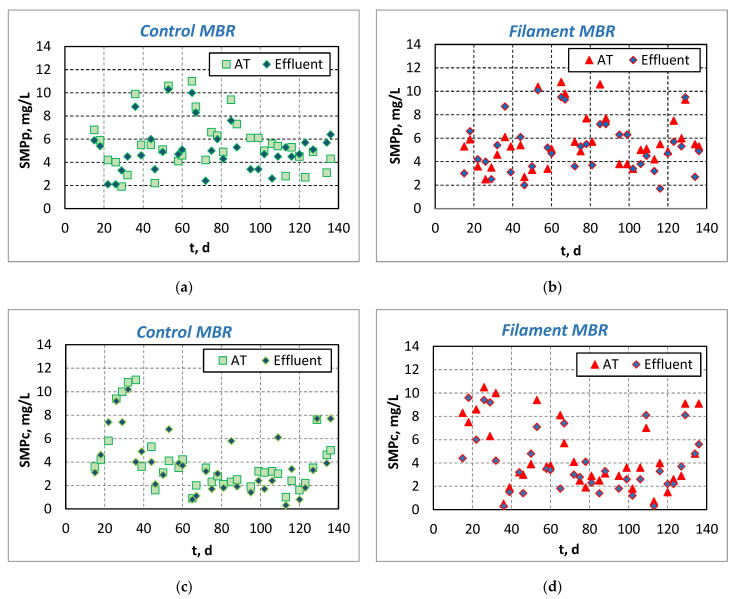
Evolution of protein and carbohydrate fraction of SMP (SMP_p_ and SMP_c_, respectively) in the aeration tank (AT) and in the effluent of the pilot-scale MBR: (**a**) SMP_p_ in the Control-MBR, (**b**) SMP_p_ in the Filament-MBR, (**c**) SMP_c_ in the Control-MBR and (**d**) SMP_c_ in the Filament-MBR.

**Figure 4:**
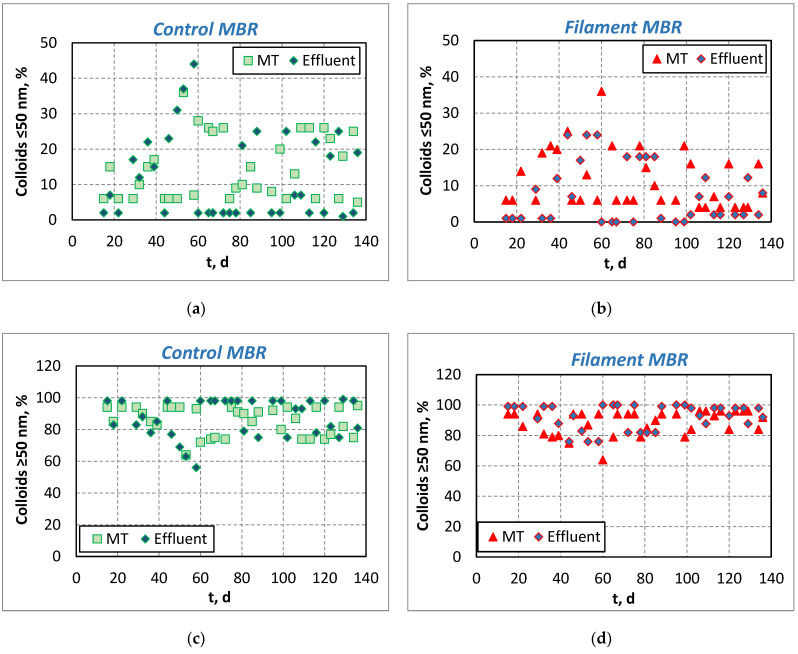
Evolution of colloids in the membrane tank (MT) and in the effluent of the pilot-scale MBR: (**a**) colloids with size ≤50 μm in the Control-MBR, (**b**) colloids with size ≤50 μm in the Filament-MBR, (**c**) colloids with size ≥50 μm in the Control-MBR and (**d**) colloids with size ≥50 μm in the Filament-MBR.

Figure 2 presents the evolution of the TMP, FI index and temperature in both pilot-MBRs. As shown in Figure 2a, during the first 80 days of operation, i.e., during the long-term fouling period (Stage 2), TMP in both pilot-MBRs was much lower than the maximum TMP (i.e., 400 mbar), considering the employed membrane module. It should be stated that, prior to the main experiments, preliminary tests were conducted which included the application of different F/M ratios and concentrations of Mixed Liquor Suspended Solids (MLSS) and DO, in order to test the MBR system and ensure its smooth operation. However, during the preliminary test operation of Filament-MBR, the applied low F/M ratio, low MLSS concentration and high DO concentration increased the amount of colloids in the mixed liquor, resulting in the fouling of the membrane (data not shown). Therefore, the membrane module was chemically cleaned, according to the instructions provided by the membrane manufacturer. In addition, during the first 15 operation days of the biomass acclimatization period, several maintenance actions were taken in order to overcome the encountered operating problems and ensure the smooth operation of all the employed equipment (pumps, blowers, automation systems, etc.). Thus, TMP, FI and the concentration of SMP and colloids, which were measured during the first 15 days, are not considered to be representative of the pilot MBRs operation and are not presented in Figure 2, Figure 3 and Figure 4. As shown in Figure 2a, during the first 80 days of operation, TMP in the Control-MBR was lower than TMP in the Filament-MBR, which had already undergone a chemical cleaning cycle. Apparently, the difference in TMP between the two pilots corresponds to the irrecoverable fouling, which was not removed after the chemical cleaning of the membrane in the Filament-MBR. However, by the end of Stage 2, TMP for both pilots tends to coincide and, during the stage of TMP jump (Stage 3), the observed TMP trend was reversed: TMP in the Filament-MBR was lower than TMP in the Control-MBR.

Careful observation of the Filament Index values (Figure 2b) and the acquired sludge images (Figure 2c) suggests that the aforementioned change in the TMP behavior is closely related to the occurred changes in sludge biology/flocculation and, more specifically, in the development of filamentous microorganisms.

As shown in Figure 2b, after the first 80 operation days, when FI was 1 in both pilots, FI started to increase and was continuously higher in the Filament-MBR until the termination of MBR operation. Figure 2c presents indicative sludge images which correspond to the final stage of TMP evolution (Stage 3) for both pilots. It is understood that, since the number of filamentous microorganisms in the Filament-MBR was slightly higher than their number in the Control-MBR, the respective TMP difference was relatively low. According to Banti et al. [9], a moderate population of filamentous microorganisms (FI = 2–3) is possibly developed when DO = 0.5 ± 0.3 mg/L and F/M = 0.4–0.5 g COD/g MLSS∙d. In the present study, although the DO concentration was controlled at the aforementioned value, the low F/M ratio (0.1 g COD/g MLSS∙d) in the filament tank due to high bio-absorption of organic matter in the denitrification tank (please see also Figure S2 in Supplementary Material) did not favour the development of filaments. Therefore, the increase in filament population after the 100th day of MBR operation can be attributed to low temperature, as FI started to increase when the temperature of mixed liquor decreased to <15 °C. This is in accordance with several research studies which associate the development of filamentous microorganisms with a lower sludge temperature [16,17].

#### 3.1.2. Investigation of Foulants—Evolution of SMP and Colloids

The almost complete bio-absorption of organic compounds that was observed in the effluent of the denitrification tanks of both pilot-scale MBRs, where the soluble COD was found equal to 23 ± 9 mg/L at the Control-MBR and 25 ± 10 mg/L at the Filament-MBR, resulted in a low F/M ratio for the subsequent treatment tanks and, therefore, in starving conditions for the growth of filamentous microorganisms. As a result, the SMP concentration was ranged at very low values, in contrast to the usual MBRs that do not incorporate a denitrification process, where the SMP proteins (SMP_p_) were found to be as high as 82 ± 20 mg/L and SMP carbohydrates (SMP_c_) as high as 20 ± 9 mg/L, according to other researchers [18]. Specifically, the average value of SMP_p_ in the Aeration Tank (AT) and in the effluent of Control-MBR was 5.5 ± 2.3 mg/L and 5.2 ± 2.0 mg/L, respectively (Figure 3a). SMP proteins were ranged at similarly low levels at the Filament-MBR, where they ranged mainly at 5.7 ± 2.2 mg/L and 5.5 ± 2.2 mg/L in the AT and in effluent, respectively (Figure 3b). The SMP_c_ also ranged at rather low values (4.0 ± 2.7 mg/L and 3.9 ± 2.5 mg/L) in the AT and in the effluent of Control-MBR (Figure 3c), and 4.7 ± 2.9 mg/L and 4.0 ± 2.6 mg/L in the AT and in the effluent of Filament-MBR, respectively (Figure 3d). The low SMP values may contribute to the lower membrane fouling rate and, thus, to lower TMP values for both MBRs. The SMP in the effluent was less than the SMP in the AT and, therefore, it was verified that a small part of the SMP was kept/maintained by the membrane in the mixed liquor, while the rest could pass through the membrane in the effluent [19]. Moreover, as was observed, the change in the recirculation rate and the hydraulic retention time during the operation days 40–60 did not affect the SMP concentration. Finally, the SMP concentrations were not affected by the FI increase in the Filament-MBR.

Figure 4a,b present particle size distribution for the colloids with a size ≤50 nm that constitute basic foulants, responsible for irreversible membrane fouling, as their size is close to the membranes’ pore size for both pilot MBRs. As a result of the HRT > 6 h by Q_r_ = 2.6·Q_in_ for the greatest period of the MBRs operation (0–40 d and 60–140 d), a low concentration of colloids was observed in the mixed liquor, as well as good aggregation of sludge, also taking into account the microscopy images of Figure 2c. The increase in the recirculation rate at Q_r_ = 4.3·Q_in_ for three weeks (40–60 d) slightly increased the colloidal concentration in the mixed liquor for both MBRs. On the other hand, the increase of filament index after the initial 100 operation days in the Filament-MBR unit, contributed to the reduction of colloids in the mixed liquor, indicating that the filaments can restrain/bind the colloids onto their surface, preventing them from passing through the membrane pores and/or avoiding to block them, therefore reducing the irreversible membrane fouling [20]. Especially in the Membrane Tank (MT) of the Control-MBR, the colloids with a size ≤50 nm accounted for 15 ± 9% of the total colloids (Figure 4a) whereas, in the Filament-MBR during the initial period of 0–100 days, 13 ± 8.4% of the total colloids had a size ≤50 nm in the MT, and after the 100 operation days the colloids of size ≤50 nm in the MT reduced to 7.9 ± 5.4% (Figure 4b). Correspondingly, the reverse trends were observed for colloids with a size ≥50 nm (Figure 4c,d). 

### 3.2. Removal of Organics and Nutrients

This section investigates the performance of the pilot-scale MBR in terms of treatment efficiency. Organic matter content was assessed in terms of COD measurement, while the removal of nutrients was estimated by measuring the concentrations of total nitrogen (TN), N-NH_4_^+^, N-NO_3_^−^ and P-PO_4_^3−^. First, the processes of nitrification and denitrification are examined, with specific regard to the effect of recirculated sludge de-aeration and recirculation rate on the concentrations of N-NO_3_^−^ and N-NH_4_^+^. Then, the influent and effluent quality parameters in both pilots (Control-MBR and Filament-MBR) are presented and discussed.

#### 3.2.1. Nitrification and Denitrification—Effect of DO Diminishing in Recirculated Sludge Flow

**Figure 5:**
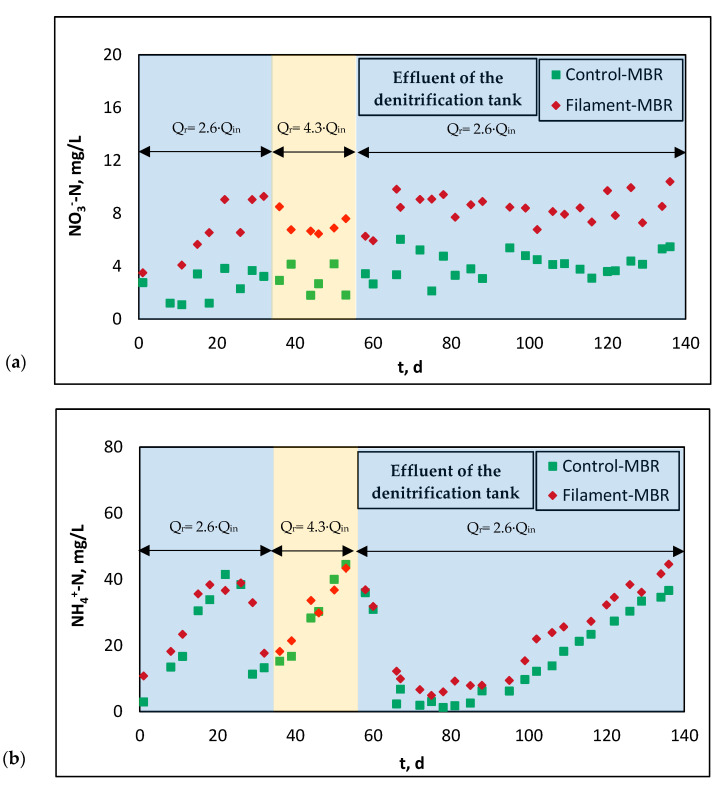
Evolution of: (**a**) N-NO_3_^−^ and (**b**) N-NH_4_^+^ concentrations in the effluent of denitrification tank for both pilot MBRs (Q_r_: recirculation rate, Q_in_: influent rate).

As mentioned in Section 2.1, a gentle stirring tank was placed in the Control-MBR prior to the denitrification tank to diminish the DO in recirculated sludge flow before the subsequent denitrification tank. Figure 5 presents the evolution of N-NO_3_^−^ and N-NH_4_^+^ concentrations in the effluent of the denitrification tank for both pilots (Control-MBR and Filament-MBR). As shown, the diminishing of DO in recirculated sludge flow below 0.5 mg/L provided an oxygen-free environment which improved the denitrification process, since lower N-NO_3_^−^ concentrations were observed in the effluent of the denitrification tank of Control-MBR (Figure 5a). On the contrary, in Filament-MBR, where the almost saturated in dissolved oxygen (DO > 6 mg/L) activated sludge, due to air scouring of the membrane, was recirculated from the membrane tank directly to the denitrification tank, and lower denitrification efficiency was achieved.

As concerns the effect of recirculation rates of Q_r_ = 2.6∙Q_in_ and 4.3∙Q_in_, it is shown that the lower recirculation rate of 2.6∙Q_in_ resulted in an improved performance, especially regarding the removal of N-NH_4_^+^ (Figure 5b). After the initial sludge acclimatization period, which lasted approximately 25 days, remarkable performance was achieved during days 20–40, when Q_r_ = 2.6∙Q_in_. The increase in recirculation rate from 2.6∙Q_in_ to 4.3∙Q_in_ during the next three weeks increased the concentration of N-NH_4_^+^ in the effluent of the denitrification tank which, in turn, resulted in higher TN and N-NO_3_^−^ in the effluent of the pilot units. However, when the recirculation rate was restored to the lowest value (2.6∙Q_in_), the concentration of N-NH_4_^+^ decreased again. This is in accordance with Tan and Ng [21], who mention that although high recycle ratios bring more nitrates back to the anoxic zone and prevent them from escaping out with the effluent, a recycle ratio of >3 deteriorates nitrogen removal efficiency due to a significant increase in DO concentration. Nonetheless, although the recirculation rate remained at this value (2.6∙Q_in_) until the end of the MBR operation, the concentration of N-NH_4_^+^ started to increase gradually after the 100th day due to decreasing temperature (<15 °C) (Figure 2b), which is known to hinder the process of denitrification and deteriorate system performance.

#### 3.2.2. Influent and Effluent Quality Parameters

**Figure 6:**
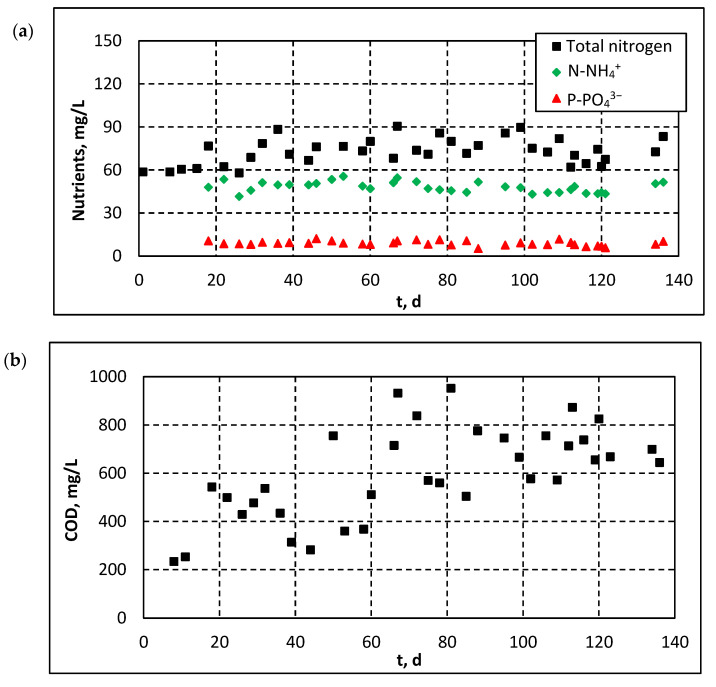
Evolution of concentrations of: (**a**) nutrients and (**b**) COD in the influent wastewater.

Figure 6 shows the influent quality parameters in the pilot-scale MBRs. As shown in Figure 6a, influent COD was gradually increased from 200 mg/L to 600–900 mg/L. This increase can be related to the seasonal variation of the local population in the region of Nea Santa, which was lower during August–September, i.e., during the initial period of MBR operation. In addition, higher water consumption during these months resulted in the dilution of the wastewater to be treated. On the contrary, remarkable stability was observed in terms of total nitrogen (TN), Ν-NH_4_^+^ and Ρ-PO_4_^3−^ concentrations in the influent. As shown in Figure 6b, these concentrations were generally maintained in the ranges of 60–90 mg/L, 40–50 mg/L and 8–12 mg/L, respectively, considered typical for municipal wastewater.

**Figure 7:**
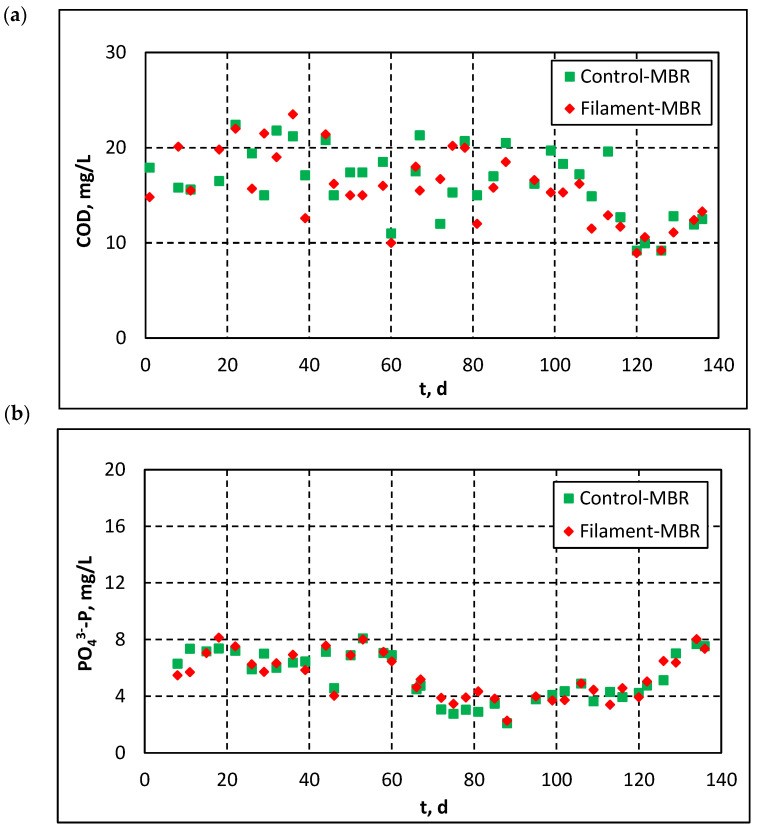
Evolution of concentrations of: (**a**) COD and (**b**) P-PO_4_^3−^ in the effluent of both pilot MBRs.

**Figure 8:**
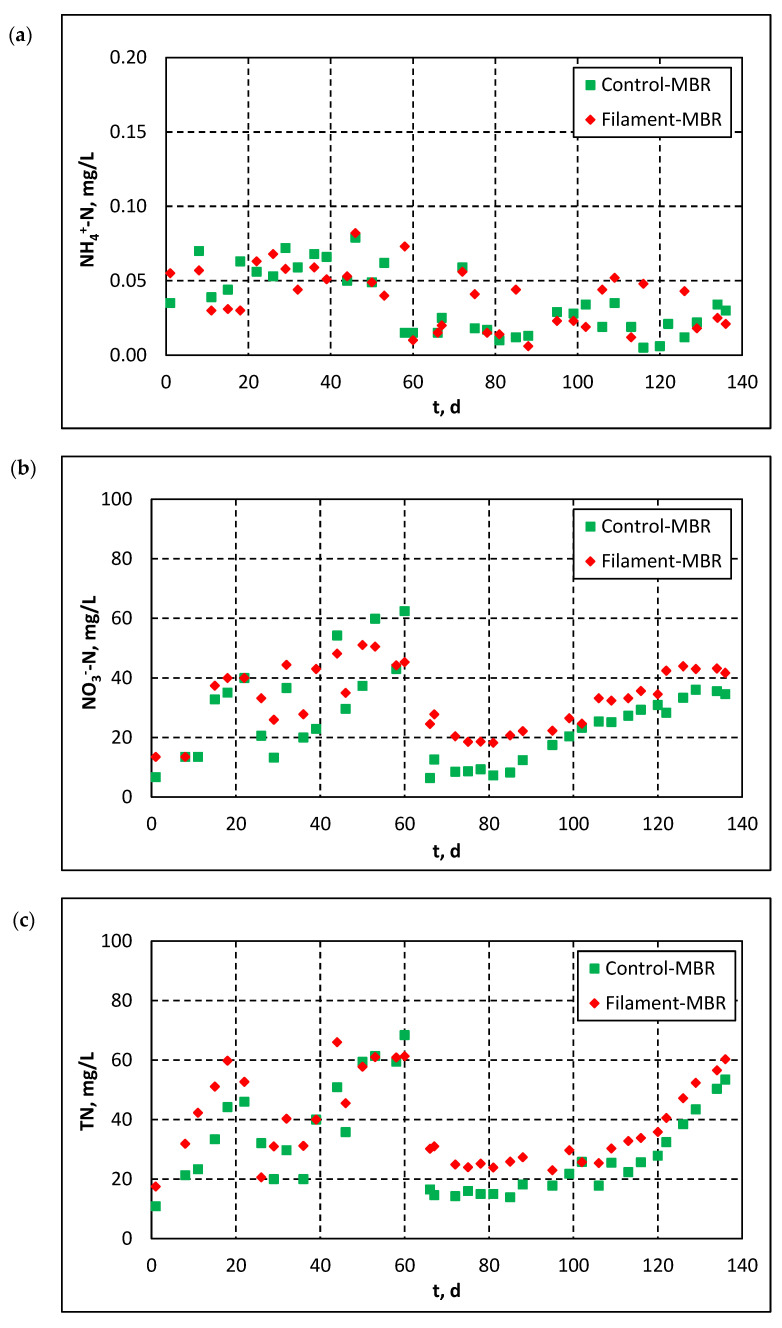
Evolution of concentrations of: (**a**) N-NH_4_^+^, (**b**) Ν-NO_3_^−^ and (**c**) total nitrogen (TN) in the effluent of both pilot MBRs.

Figure 7 and Figure 8 show the effluent quality parameters in both pilots (Control-MBR and Filament-MBR) of the pilot-scale MBR. As shown in Figure 7a, the combination of biological degradation with membrane filtration resulted in high organics removal, since COD concentration in the effluent of both pilots was generally in the range of 10–25 mg/L and occasionally decreased to <10 mg/L. On the contrary, no significant changes were observed regarding the concentration of P-PO_4_^3−^ in the effluent, which was relatively high for both pilots (Figure 7b). Ammonium was largely reduced and the concentration of N-NH_4_^+^ was decreased to <0.1 mg/L (Figure 8a). The evolution of Ν-NO_3_^−^ concentration in the effluent (Figure 8b) presents the same fluctuations, which were previously observed in the effluent of the denitrification tank (Figure 5b), depending on the employed recirculation rate. It is understood that the evolution of TN follows the same pattern (Figure 8c), since it consists mainly of Ν-NO_3_^−^. Figure 8b,c also stress the importance of recirculated sludge de-aeration: the concentrations of Ν-NO_3_^−^ and TN were higher in the effluent of the Filament-MBR, because in the recirculated sludge the DO was not diminished.

## 4. Conclusions

Although DO concentration in the filament tank of the Filament-MBR was maintained at 0.5 ± 0.3 mg/L, the low F/M ratio (0.1 g COD/g MLSS∙d) prevented the significant growth of filamentous microorganisms. However, the slight increase in filaments due to low temperature (<15 °C) reduced membrane fouling, since the observed TMP in the Filament-MBR was lower than the TMP in the Control-MBR. The almost complete bio-absorption of organic compounds in the denitrification tanks of both pilot-MBRs, which in turn led to a low F/M ratio at the subsequent tanks, allowing the SMP proteins and carbohydrates to be kept at very low values in the mixed liquor, less than 10 mg/L. Additionally, the low recirculation rate (2.6∙Q_in_) resulted in a low concentration of colloids with a size ≤50 nm (membranes pore size), accounting for 15% of the total colloids at their maximum. Moreover, the increased filamentous population at the Filament-MBR after the 100th day contributed to the further reduction of colloids in the mixed liquor at 7.9%, indicating that the filaments restrained the colloids, preventing them from passing through the membrane pores and blocking them. The low SMP and colloids values conduced beneficially to the TMP and membrane fouling reduction.

The zeroing of DO in the recirculated activated sludge stream in the Control-MBR improved the denitrification process, since lower N-NO_3_^−^ concentrations were observed in the effluent of the denitrification tank. In contrast, lower denitrification efficiency was achieved in Filament-MBR, because of DO > 6 mg/L in the recirculated activated sludge from the membrane tank which directly flowed to the denitrification tank. It was also shown that the lower recirculation rate (2.6∙Q_in_) resulted in improved performance, especially regarding the removal of N-NH_4_^+^. Finally, the combination of biological degradation with membrane filtration resulted in high organics removal and ammonium nitrification, since COD and Ν-ΝH_4_^+^ concentrations in the effluent of both pilots were generally in the range of 10–25 mg/L (occasionally <10 mg/L) and <0.1 mg/L, respectively.

## Figures and Tables

**Table 1:** Applied operating conditions and membrane characteristics in pilot-MBRs.

**Operating conditions**	
Inlet flow rate	Q_in_ = 700 L/h
Outlet (permeate) flow rate	Q_out_ = 700 L/h
Recirculation rate	Q_r_ = 1800 L/h and 3000 L/h
Flux	J = 13.5 Lm^−2^h^−1^
DO concentration	Aeration tanks: DO = 2.5 ± 0.5 mg/L
	Filament tank: DO = 0.5 ± 0.3 mg/L
	Denitrification tanks: DO < 0.2 mg/L
MLSS concentration	MLSS = 6500 mg/L
**Membrane characteristics**	
Module type	NADIR^®^ UP150 (Microdyn-Nadir GmbH)
Construction material	Polyethersulfone
Configuration	Flat sheet
Pore size	0.04 μm
Membrane area	52 m^2^
Maximum TMP filtration limit	400 mbar

## Supplementary Materials

The following are available online at https://www.mdpi.com/article/10.3390/membranes11070490/s1. Figure S1: (**a**) Engine room equipment of the MBR, (**b**) Top-view of the MBR compartment’s left side, (**c**) Top-view of the MBR compartment’s right side (A1: Gentle stirring (de-aeration) tank of Control-MBR, A2: Denitrification tank of Control-MBR, A3: Aeration tank of Control-MBR, A4: Membrane tank of Control-MBR, B1: Denitrification tank of Filament-MBR, B2: Filament tank of Filament-MBR, B3: Aeration tank of Filament-MBR, B4: Membrane tank of Filament-MBR). Figure S2: Evolution of COD concentration in the effluent of denitrification tank for both pilot MBRs. Section S1: Description of the Eikelboom method for the estimation of filaments’ population. Section S2: Description of the phenol-sulfuric acid method for the determination of SMP_c_.

## Nomenclature

*ASP*	Activated Sludge Process
*COD*	Chemical Oxygen Demand
*DO*	Dissolved Oxygen
*EPS*	Extracellular Polymeric Substances
*F/M*	Food to Microorganisms
*FI*	Filament Index
*MBR*	Membrane Bio-Reactor
*MLSS*	Mixed Liquor Suspended Solids
*Q_in_*	influent rate
*Q_out_*	filtration rate
*Q_r_*	recirculation rate
*SCADA*	Supervisory Control and Data Acquisition
*SMP*	Soluble Microbial Products
*SMP_c_*	carbohydrate fraction of SMP
*SMP_p_*	protein fraction of SMP
*SRT*	Solids Retention Time
*TMP*	Trans-Membrane Pressure
*TN*	Total Nitrogen

## References

[1] Nam K., Heo S., Rhee G., Kim M., Yoo C. (2021). Dual-objective optimization for energy-saving and fouling mitigation in MBR plants using AI-based influent prediction and an integrated biological-physical model. J. Membr. Sci..

[2] Yin X., Li X., Hua Z., Ren Y. (2020). The growth process of the cake layer and membrane fouling alleviation mechanism in a MBR assisted with the self-generated electric field. Water Res..

[3] Gkotsis P., Zouboulis A., Mitrakas M. (2020). Using Additives for Fouling Control in a Lab-Scale MBR; Comparing the Anti-Fouling Potential of Coagulants, PAC and Bio-Film Carriers. Membranes.

[4] Martins A.M., Pagilla K., Heijnen J.J., van Loosdrecht M.C. (2004). Filamentous bulking sludge—A critical review. Water Res..

[5] Meng F., Zhang H., Yang F., Li Y., Xiao J., Zhang X. (2006). Effect of filamentous bacteria on membrane fouling in submerged membrane bioreactor. J. Membr. Sci..

[6] Meng F., Yang F. (2007). Fouling mechanisms of deflocculated sludge, normal sludge, and bulking sludge in membrane bioreactor. J. Membr. Sci..

[7] Li J., Li Y., Ohandja D.-G., Yang F., Wong F.-S., Chua H.-C. (2008). Impact of filamentous bacteria on properties of activated sludge and membrane-fouling rate in a submerged MBR. Sep. Purif. Technol..

[8] Wang Z., Wang P., Wang Q., Wu Z., Zhou Q., Yang D. (2010). Effective control of membrane fouling by filamentous bacteria in a submerged membrane bioreactor. Chem. Eng. J..

[9] Banti D.C., Karayannakidis P.D., Samaras P., Mitrakas M.G. (2017). An innovative bioreactor set-up that reduces membrane fouling by adjusting the filamentous bacterial population. J. Membr. Sci..

[10] Gkotsis P., Lemonidis G., Mitrakas M., Pentedimos A., Kostoglou M., Zouboulis A. (2020). Quantifying the Effect of COD to TN Ratio, DO Concentration and Temperature on Filamentous Microorganisms’ Population and Trans-Membrane Pressure (TMP) in Membrane Bio-Reactors (MBR). Processes.

[11] Hao L., Liss S., Liao B. (2016). Influence of COD:N ratio on sludge properties and their role in membrane fouling of a submerged membrane bioreactor. Water Res..

[12] You S., Sue W. (2009). Filamentous bacteria in a foaming membrane bioreactor. J. Membr. Sci..

[13] Eikelboom D.H. (2000). Process Control of Activated Sludge Plants by Microscopic Investigation.

[14] Dubois M., Gilles K.A., Hamilton J.K., Rebers P.A., Smith F. (1956). Colorimetric Method for Determination of Sugars and Related Substances. Anal. Chem..

[15] Hartree E. (1972). Determination of protein: A modification of the lowry method that gives a linear photometric response. Anal. Biochem..

[16] Pajdak-Stós A., Fiałkowska E. (2012). The influence of temperature on the effectiveness of filamentous bacteria removal from activated sludge by rotifers. Water Environ. Res..

[17] Guo J., Peng Y., Wang S., Yang X., Wang Z., Zhu A. (2012). Stable limited filamentous bulking through keeping the competition between floc-formers and filaments in balance. Bioresour. Technol..

[18] Banti D., Mitrakas M., Fytianos G., Tsali A., Samaras P. (2020). Combined Effect of Colloids and SMP on Membrane Fouling in MBRs. Membranes.

[19] Banti D.C., Samaras P., Tsioptsias C., Zouboulis A., Mitrakas M., Dimitra B.C., Petros S., Costas T., Anastasios Z., Manassis M. (2018). Mechanism of SMP aggregation within the pores of hydrophilic and hydrophobic MBR membranes and aggregates detachment. Sep. Purif. Technol..

[20] Christensen M., Niessen W., Sørensen N.B., Hansen S.H., Jørgensen M.K., Nielsen P.H. (2018). Sludge fractionation as a method to study and predict fouling in MBR systems. Sep. Purif. Technol..

[21] Tan T.W., Ng H.Y. (2008). Influence of mixed liquor recycle ratio and dissolved oxygen on performance of pre-denitrification submerged membrane bioreactors. Water Res..

