# Supplementary material for: Effect of Operating Conditions on Membrane Fouling in Pilot-Scale MBRs: Filaments Growth, Diminishing Dissolved Oxygen and Recirculation Rate of the Activated Sludge
Source: Membranes (Basel). 2021 Jun 29;11(7):490. doi: 10.3390/membranes11070490 (PMC8303956; doi:10.3390/membranes11070490)
Supplement: Supplementary file 1 [file membranes-11-00490-s001.zip › membranes-1266679-supplementary.pdf]

SUPPLEMENTARY DATA

Effect of Operating Conditions on Membrane Fouling in Pilot-Scale MBRs; FILAMENTS Growth, Diminishing Dissolved Oxygen and Recirculation Rate of the Activated Sludge

Petros Gkotsis [1], Dimitra Banti [2], Anastasia Pritsa [3], Manassis Mitrakas [3], Petros Samaras [2], Efrosini Peleka[1] and Anastasios Zouboulis [1,*]

[1] Laboratory of Chemical and Environmental Technology, Department of Chemistry, Faculty of Sciences, Aristotle University of Thessaloniki, GR-54124, Thessaloniki, Greece; petgk@chem.auth.gr; peleka@chem.auth.gr, zoubouli@chem.auth.gr
[2] Laboratory of Technologies of Environmental Protection and Utilization of Food By-Products, Department of Food Science and Technology, International Hellenic University, GR-57400, Thessaloniki, Greece; bantidim@gmail.com; samaras@ihu.gr
[3] Analytic Chemistry Laboratory, Department of Chemical Engineering, School of Engineering, Aristotle University of Thessaloniki, GR-54124, Thessaloniki, Greece; pritsa.anastasia@gmail.com; manasis@eng.auth.gr
* Correspondence: zoubouli@chem.auth.gr; Tel.: +30 2310-997-794

Materials and methods

Pilot-scale MBR configuration and operation

(a)

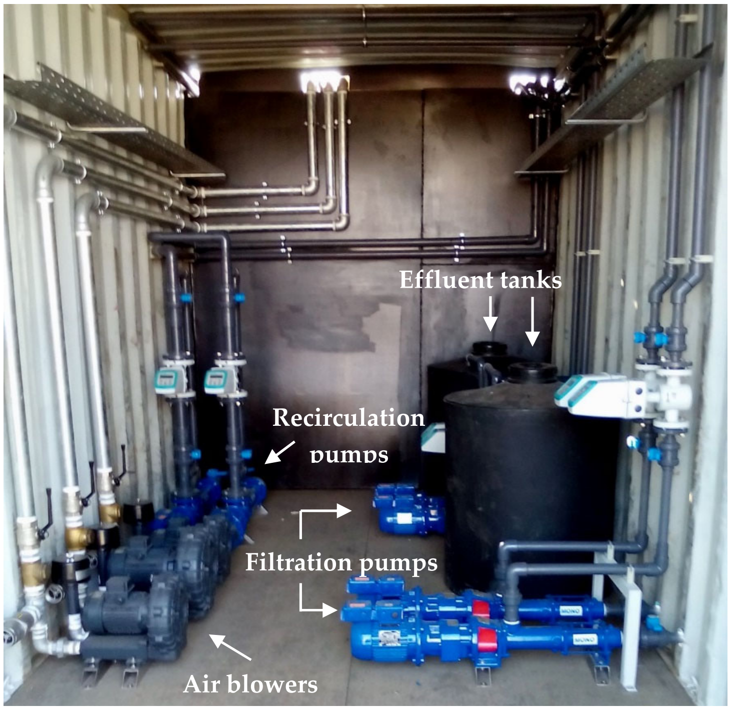

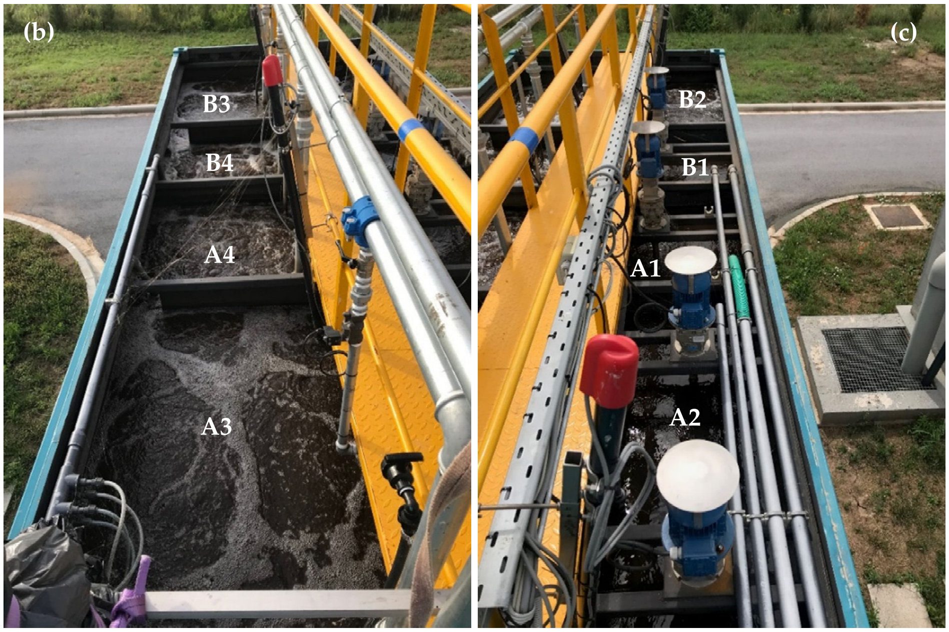

Figure S1. **(a)** Engine room equipment of the MBR, **(b)** Top-view of the MBR compartment's left side, **(c)** Top-view of the MBR compartment's right side (A1: Gentle stirring (de-aeration) tank of Control-MBR, A2: Denitrification tank of Control-MBR, A3: Aeration tank of Control-MBR, A4: Membrane tank of Control-MBR, B1: Denitrification tank of Filament-MBR, B2: Filament tank of Filament-MBR, B3: Aeration tank of Filament-MBR, B4: Membrane tank of Filament-MBR).

Analytical techniques and protocols

S1. Description of the Eikelboom method for the estimation of filaments' population

According to this method, a scale of 0-5 is used from none to infinite filaments. Between the consecutive FI classes, there is a difference of approximately a factor of 10. The FI is determined by comparing the microscopic images of the sludge, at a low magnification, with a series of reference photos of the various FI classes. The mixed liquor receives the FI value of the photo which best corresponds to the number of filamentous microorganisms in the microscopic image. When FI = 1 or 2, the effect of filamentous microorganisms on the settling velocity of the sludge is slight. When FI = 3, the settling properties are often significantly deteriorated and when FI > 3 bulking usually occurs.

S2. Description of the phenol-sulfuric acid method for the determination of SMP_c

The principle of this method is that carbohydrates, when dehydrated by reaction with concentrated sulfuric acid, produce furfural derivatives. Further reaction between furfural derivatives and phenol develops a detectible colour. A short description of the standard procedure is following: 1 mL of a carbohydrate solution was mixed with 1 mL of wt. 5% aqueous solution of phenol in a test tube. Subsequently, 5 mL of concentrated H_2SO_4 were added rapidly to the mixture. After allowing the test tubes to stand for 10 min, they were vortexed for 30 s and placed for 20 min in a water bath at room temperature for colour development. Then, light absorption at 480 nm was recorded on a spectrophotometer. Reference solutions were prepared in identical manner as aforementioned, except that the 1 mL of carbohydrate was replaced by glucose.

Results and discussion

Bio-adsorption of organic matter in the denitrification tanks

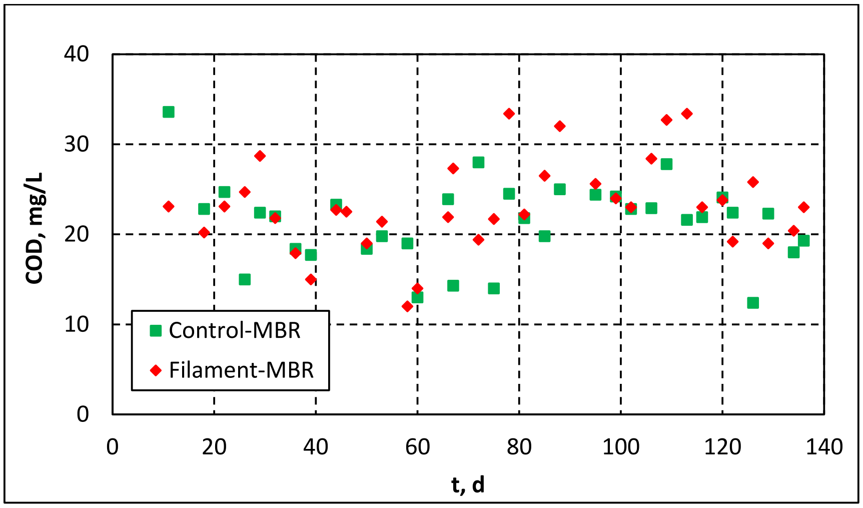

Figure S2. Evolution of COD concentration in the effluent of denitrification tank for both pilot MBRs.
